# Long-Term Follow-Up and Predictors of Functional Outcome after Surgery for Spinal Meningiomas: A Population-Based Cohort Study

**DOI:** 10.3390/cancers13133244

**Published:** 2021-06-29

**Authors:** Jenny Pettersson-Segerlind, Alexander Fletcher-Sandersjöö, Charles Tatter, Gustav Burström, Oscar Persson, Petter Förander, Tiit Mathiesen, Jiri Bartek, Erik Edström, Adrian Elmi-Terander

**Affiliations:** 1 Department of Neurosurgery, Karolinska University Hospital, 171 64 Stockholm, Sweden; jenny.pettersson-segerlind@sll.se (J.P.-S.); alexander.fletcher-sandersjoo@sll.se (A.F.-S.); charles.tatter@sll.se (C.T.); gustav.liu-burstrom@sll.se (G.B.); oscar.persson@sll.se (O.P.); petter.forander@sll.se (P.F.); jiri.bartek@sll.se (J.B.J.); erik.edstrom@sll.se (E.E.); 2 Department of Clinical Neuroscience, Karolinska Institutet, 171 77 Stockholm, Sweden; tiit.illimar.mathiesen@regionh.dk; 3 Department of Neurosurgery, Rigshospitalet, 2100 Copenhagen, Denmark

**Keywords:** meningioma, spine, spinal meningioma, elderly, age, surgery, neurosurgery

## Abstract

**Simple Summary:**

Spinal meningiomas are the most common adult primary intradural spinal tumors. While mostly benign, they may give rise to spinal cord compression with acute or chronic neurologic dysfunction. The primary treatment is surgical resection. Previous studies, limited by small sample sizes and short follow-up times, report that histopathological grade, tumor localization and size affect outcomes following surgery. In this population-based cohort study, we retrospectively reviewed 129 cases of surgically treated spinal meningiomas to assess postoperative complications, long-term clinical and radiological outcomes, predictors of neurological improvement and potential differences between elderly and non-elderly patients. Our median follow-up time was 8.2 years. We found that surgery was associated with significant neurological improvement. There was no significant difference in postoperative complications, tumor control or neurological improvement between elderly and non-elderly. Shorter time from diagnosis to surgery, larger tumor size and spinal cord compression predicted postoperative outcomes.

**Abstract:**

Spinal meningiomas are the most common adult primary spinal tumor, constituting 24–45% of spinal intradural tumors and 2% of all meningiomas. The aim of this study was to assess postoperative complications, long-term outcomes, predictors of functional improvement and differences between elderly (≥70 years) and non-elderly (18–69 years) patients surgically treated for spinal meningiomas. Variables were retrospectively collected from patient charts and magnetic resonance images. Baseline comparisons, paired testing and regression analyses were used. In conclusion, 129 patients were included, with a median follow-up time of 8.2 years. Motor deficit was the most common presenting symptom (66%). The median time between diagnosis and surgery was 1.3 months. A postoperative complication occurred in 10 (7.8%) and tumor growth or recurrence in 6 (4.7%) patients. Surgery was associated with significant improvement of motor and sensory deficit, gait disturbance, bladder dysfunction and pain. Time to surgery, tumor area and the degree of spinal cord compression significantly predicted postoperative improvement in a modified McCormick scale (mMCs) in the univariable regression analysis, and spinal cord compression showed independent risk association in multivariable analysis. There was no difference in improvement, complications or tumor control between elderly and non-elderly patients. We concluded that surgery of spinal meningiomas was associated with significant long-term neurological improvement, which could be predicted by time to surgery, tumor size and spinal cord compression.

## 1. Introduction

Spinal meningiomas are intradural extramedullary tumors that originate from the meningothelial cells in the leptomeninges of the spinal cord. They have an age-adjusted incidence of 0.33 per 100,000 population, making them the most common adult primary spinal tumor [1]. They constitute 25–45% of all spinal tumors and 2% of all meningiomas. The male to female ratio is 1:4.5 and they are more prevalent in the elderly population, with a peak incidence between the 6th and 8th decade of life [1,2]. While most spinal meningiomas are World Health Organization (WHO) grade 1 [3,4,5] (benign and typically with a low Ki 67 proliferation index (MIB1-index [6])), they may give rise to spinal cord compression with acute or chronic neurologic dysfunction [3,7]. The primary treatment for spinal meningiomas is surgical resection [7], and gross total resection (Simpson grade 1–3) can often be achieved with sustained or improved neurological function [6,8,9,10,11]. A number of previous studies report conflicting data on the effects of tumor size, histopathological grade and preoperative neurological impairment on outcome following spinal meningioma surgery [2,10,11,12,13,14,15,16,17,18,19,20]. Moreover, studies have suffered from factors such as limited sample size [2,13,15,16,17,18,19,21,22] and short follow-up time [2,15,18,19]. In addition, even though the incidence of spinal meningiomas is highest in the elderly population, there may be a reluctance to operate on these patients due to an expected higher risk of adverse events and poor outcomes [1,22,23,24,25,26,27,28]. Available studies on this topic have been limited by low patient numbers and lack of a younger control group [21,22,29] (Table S1).

In this population-based cohort study, we retrospectively reviewed 129 cases of surgically treated spinal meningiomas in order to assess baseline data, postoperative complications, long-term clinical and radiological outcomes, predictors of neurological improvement and potential differences between elderly and non-elderly patients.

## 2. Materials and Methods

### 2.1. Patient Selection and Study Setting

All adult patients (≥18 years) who were surgically treated for a spinal meningioma 2005–2017 were eligible for inclusion. Patients with neurological comorbidities were not excluded. The study hospital is a publicly funded and owned tertiary care center serving a region of roughly 2.3 million inhabitants, and the only neurosurgical center in the region. Patients were identified using the surgical management software Orbit (Evry Healthcare Systems, Solna, Sweden). Medical records and imaging data from digital hospital charts were retrospectively reviewed using the health record software TakeCare (CompuGroup Medical Sweden AB, Farsta, Sweden). The study was approved by the Regional and National Ethical Review Board (Dnr: 2016/1708-31/4 and 2020-00192).

### 2.2. Surgical Technique and Follow-Up Routine

Prior to surgery, the spinous process of the vertebra adjacent to the tumor (if thoracic or lumbar) was identified using computed tomography guidance and marked with the injection of a sterile carbon suspension. For cervical tumors, levels were identified with fluoroscopy. With the patient in the prone position, a posterior midline approach was performed. Laminectomy was conducted using an ultrasonic bone scalpel (from 2012 to 2017) (Misonix Inc., Farmingdale, NY, USA) or a high-speed drill with a diamond-coated bur and Kerrison rongeurs (from 2005 to 2011). Under the microscope, the dura was incised and held open by sutures, allowing exposure of the tumor. The arachnoid was dissected sharply, and the cranial and caudal poles of the tumor were identified. The tumor was then coagulated to reduce size and blood supply and dissected from surrounding structures. When possible, the dural attachment site was meticulously coagulated (Simpson grade 2), but never excised to achieve a Simpson grade 1 resection. Neurophysiological monitoring was not used. In all cases, watertight dural closure was performed. Duraplasty was performed when deemed appropriate. When laminoplasty was performed, the laminae were repositioned using microplates (CMF Medicon Surgical Inc., Jacksonville, FL, USA). The soft tissues were then sutured in layers to close the wound. For WHO grade 1 tumors, the institutional follow-up routine consisted of clinical assessment 3–6 months after surgery at the outpatient department as well as magnetic resonance imaging (MRI) at 3 months and 1, 3, 5, 7 and 10 years after surgery. For WHO grade 2 tumors, or tumors where intraoperative conditions may suggest the risk of early recurrence, MRI was performed every 6 months for the first year and then annually as long as no recurrence or growth was identified.

### 2.3. Variables

**Figure 1:**
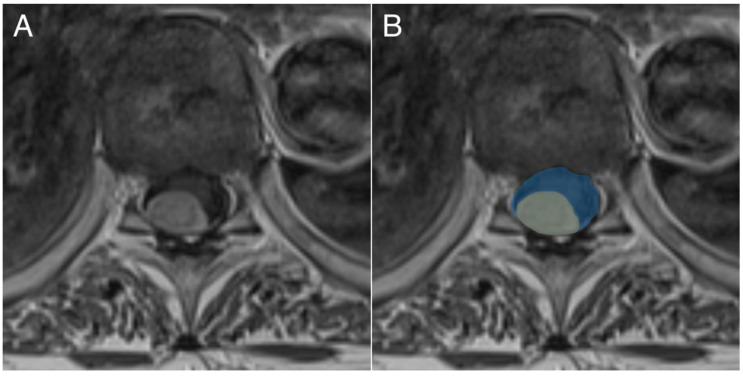
Illustration depicting how cross-sectional tumor area (yellow) and spinal canal area (blue) were calculated from preoperative magnetic resonance images (MRI). (**A**) shows the original MRI, and (**B**) shows the annotation of the tumor and spinal canal area.

The following pre-operative data were collected: age, sex, American Society of Anesthesiologists (ASA) class, prior radiotherapy, prior spinal surgery, neurological symptoms, modified McCormick Scale (mMCs) [30] (Table 1), tumor location, tumor area, spinal canal area and spinal cord compression. Age was classified into elderly (≥70 years) and non-elderly (18–69 years), in accordance with previous studies [21,22,24,29,31]. The cross-sectional area of the spinal canal and tumor was calculated from axial contrast enhanced T1-weighted MRI slices where the tumor diameter was the largest (Figure 1). Spinal cord compression was defined as the percentage of the spinal canal occupied by tumor, based on the cross-sectional area outlined above.

The following pre and postoperative data were collected: time from diagnosis to surgery, laminectomy range, Simpson grade, MIB1-index, World Health Organization (WHO) grade, adjuvant treatment, postoperative complications, follow-up time, long-term tumor growth and/or recurrence, long-term neurological symptoms, change in mMCs, mortality and cause of death. Tumor growth was defined as the radiological growth of a tumor remnant following subtotal resection, while tumor recurrence was defined as the appearance of a new spinal meningioma following total resection. Improvement in mMCs was calculated by subtracting the preoperative mMCs from each patient’s postoperative value (obtained at the 3–6 months follow-up), with improvement defined as a decrease in mMCs by at least one point. All histopathological analyses were performed at the Department of Pathology, Karolinska University Hospital, Stockholm, Sweden. MIB-1 indexes were analyzed manually. Patients were classified according to WHO criteria from 2007. However, as no patients showed signs of spinal cord invasion, the grading is consistent with the 2016 WHO classification of meningiomas [32].

### 2.4. Statistics

The Shapiro-Wilk test was used to evaluate the normality of the data. As all continuous data significantly deviated from a normal distribution pattern (Shapiro-Wilk test *p* value < 0.05), it is presented as a median (range) and categorical data as numbers (proportion). For continuous variables, the Mann-Whitney U test was used for between-group comparisons, and the Wilcoxon matched-pairs signed-ranks test for within-group comparisons. For categorical data, the chi-square test was used for between-group comparisons and McNemar’s test for within-group comparisons. A univariable and stepwise multivariable logistic regression model was employed to identify predictors of improved postoperative mMCs. Variables were included in the multivariable regression if they had a *p*-value < 0.1 in the univariable model. Listwise deletion was used to handle missing data. All analyses were conducted using the statistical software program R version 4.0.5, utilizing the graphical interface RStudio^®^ (Boston, MA, USA) [33]. Statistical significance was set at *p* < 0.05.

## 3. Results

### 3.1. Baseline and Treatment Data

**Figure 2:**
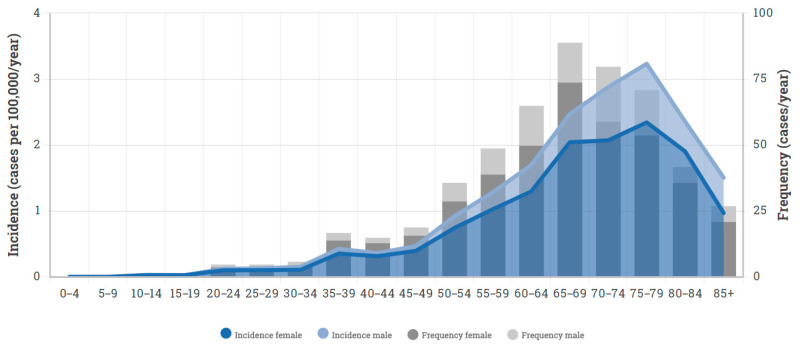
Stacked bar chart and density plot, using data from the Swedish national cancer registry, highlighting the incidence and frequency of spinal meningiomas 2005–2017.

In total, 138 patients met the inclusion criteria. Of these, 9 were excluded due to missing data and the remaining 129 were included in the study. The median age was 65 years and 106 (82%) were female (Figure 2). The median preoperative mMCs was 2 (range 1–4), and motor deficit was the most common presenting symptom (*n* = 85; 66%), followed by sensory deficit (*n* = 82; 64%) and gait disturbance (*n* = 79; 61%). The most common tumor localization was at the thoracic level (*n* = 89; 69%), and the median cross-sectional tumor area was 1.4 cm^2^ (range 0.2–4.5) with a median spinal cord compression of 69% (range 12–89%). Eight patients (6%) had a concurrent cranial meningioma (Table 2).

The median time between diagnosis and surgery was 1.3 months (range 0.03–36). A Simpson grade 2 resection was achieved in 92 (71%) cases, with those that remained being either grade 3 (*n* = 17; 13%) or 4 (*n* = 20; 16%). Most of the tumors were WHO grade 1 (*n* = 127; 98%), and the median MIB1-index was 4.5% (range 0–20) (Table 3). One patient, with an atypical metastatic meningioma treated with Simpson grade 4 resection, received postoperative radiotherapy but passed away shortly thereafter from a pulmonary metastasis.

### 3.2. Outcome Data

The median follow-up time was 8.2 years (range 0.3–16). It is of note that the shortest follow-up time of 0.3 years was an outlier patient with an atypical metastatic meningioma who passed away from a pulmonary metastasis shortly after surgery, and our second shortest follow-up time was 1.7 years. Forty-six patients were followed for more than 10 years and 16 of these were within the elderly cohort. Postoperative complications occurred in 10 (7.8%) patients, of which wound infection was the most common (*n* = 3; 2.3%). Four patients (3.1%) were reoperated due to local tumor growth (*n* = 1), tumor recurrence (*n* = 1), tethered spinal cord (*n* = 1) or wound infection (*n* = 1). Long-term mortality was 21% (*n* = 27), with a median age at death of 82 years (range 61–98). No deaths occurred within 3 months after surgery, and no patients died from any complications related to the surgical procedure (Table 4).

**Figure 3:**
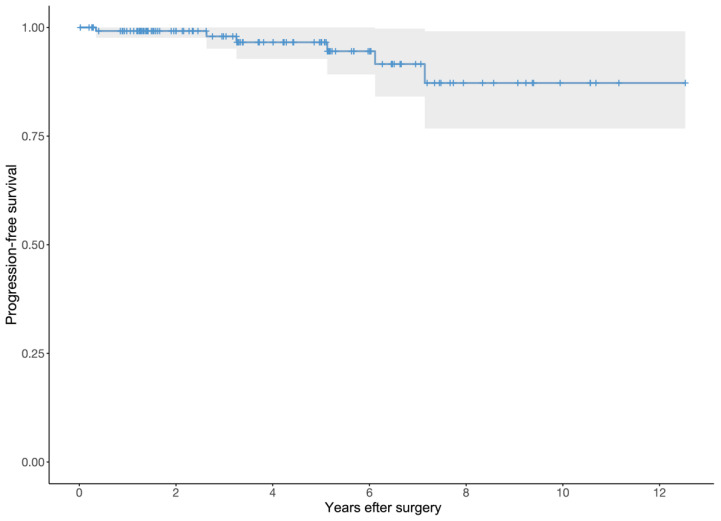
Kaplan-Meier survival curve of tumor recurrence or growth following surgical resection of spinal meningiomas.

Tumor growth occurred in four patients (3.1%), all Simpson grade 4 resections, of which 3 were left in close proximity to a nerve root. In the fourth case, a tumor remnant was left as it infiltrated the pia mater. For these cases, tumor growth occurred 4 months (MIB 7%), 3.2 years (MIB 3%), 6.1 years (MIB 3%) and 7.1 years (MIB 5%) after surgery, respectively. One of the patients was reoperated upon due to renewed spinal cord compression and radicular pain, while the three remaining patients only had minor growths with no new neurological symptoms. Tumor recurrence, following Simpson grade 2 resections, occurred in two other patients (1.6%). One of these recurrences occurred after 2.6 years (MIB 15–20%), and this patient underwent reoperation due to progressing motor deficit. The other recurrence occurred after 5.1 years (MIB 1–2%), but did not cause any significant spinal cord compression and was therefore not reoperated upon. Conservatively treated patients with tumor growth or recurrence were naturally subjected to further radiological follow-up. A Kaplan-Meier survival curve of tumor recurrence and growth is provided as Figure 3.

### 3.3. Functional Outcome

Paired testing showed that surgery was associated with a significant improvement in motor deficit, sensory deficit, gait disturbance, bladder dysfunction and pain (*p* < 0.001 for all analyses). Of these, bladder dysfunction was the symptom that most often improved (76%), followed by pain (53%) and gait disturbance (51%) (Table 5). On a group level, 61 patients (47%) improved in their mMCs following surgery, with the others remaining unchanged (*n* = 66, 51%) or worsening (*n* = 2, 1.5%). It should be noted that 33 patients were mMCs 1 prior to surgery and could thus not improve. Of the two cases that were worse after surgery (i.e., increased mMCs), one was caused by progression in an underlying multiple sclerosis, unfortunately not related to the surgery. The other patient was a case of a calcified meningioma with anterior attachment at the level of conus medularis (Th12). This patient had a slight weakness of the right lower limb preoperatively that deteriorated to a paraparesis immediately after surgery, and then improved slightly to a monoparesis of the right lower limb at follow-up. This patient had an intramedullary high signal intensity on T2-weighted postoperative MRI, a sign of spinal cord injury.

### 3.4. Elderly vs. Non-Elderly Patients

**Figure 4:**
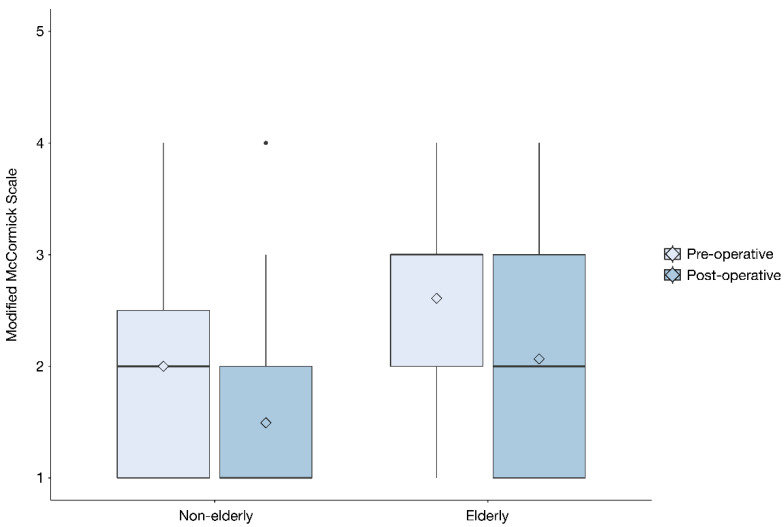
Box plot depicting pre- and postoperative modified McCormick scale for the elderly and non-elderly cohort. The dot represents an outlier.

**Figure 5:**
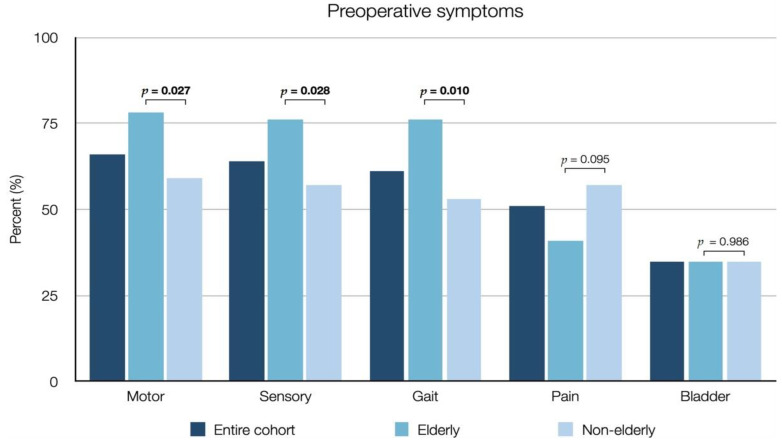
Bar chart showing preoperative symptoms in elderly (≥70 years) and non-elderly (18–69 years) patients, as well as the entire cohort, who underwent surgical resection of spinal meningiomas. Bold text indicates a statistically significant correlation (*p* < 0.05).

**Figure 6:**
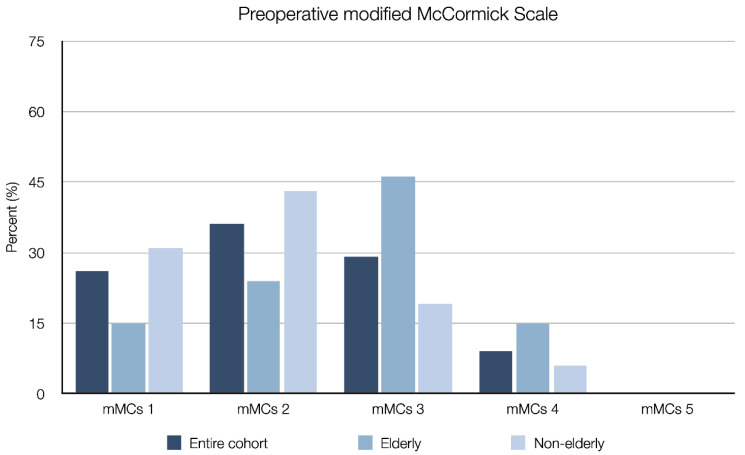
Bar chart showing preoperative modified McCormick scale in elderly (≥70 years) and non-elderly (18–69 years) patients, as well as the entire cohort, who underwent surgical resection of spinal meningiomas.

Forty-six (36%) patients were classified as elderly. The median age for the elderly group was 76 years (range 70–94), as compared to 58 years (range 23–69) in the non-elderly group. Compared to the non-elderly group, elderly patients presented with a significantly higher ASA class (median 3 vs. 2, *p* = 0.023), a higher preoperative mMCs (median 3 vs. 2, *p* < 0.001) (Figure 4) and more frequently suffered from motor deficits (78% vs. 59%, *p* = 0.027), sensory deficits (76% vs. 57%, *p* = 0.028) and gait disturbances (76% vs. 53%, *p* = 0.010) (Table 2, Figure 5 and Figure 6). Elderly patients also had a significantly shorter time from diagnosis to surgery (median 0.5 vs. 1.8 months, *p* = 0.005). There was no significant difference between the elderly and non-elderly groups with regard to preoperative radiological data, Simpson grade, WHO grade, MIB1-index (Table 3), postoperative improvement in mMCs or the frequency of postoperative complications (Table 4).

### 3.5. Predictors of Improved Functional Outcome

In the univariable logistic regression predicting postoperative improvement in mMCs, a significant association was seen for time to surgery (*p* < 0.001, OR (odds ratio) 0.86, 95% CI 0.76–0.94, R^2^ = 0.141), tumor area (*p* = 0.030, OR 1.73, 95% CI 1.00–3.17, R^2^ = 0.053) and spinal cord compression (*p* < 0.001, OR 1.03, 95% CI 1.01–1.06, R^2^ = 0.137) (Table 6). Of these, spinal cord compression remained independently associated with mMCs improvement (*p* = 0.002) in the step-down multivariable model (Table 7).

Subdivided by age category, time to surgery (*p* < 0.001), tumor area (*p* = 0.014) and spinal cord compression (*p* = 0.002) were all significant in univariable analysis for the non-elderly cohort (Table S2), but not in the elderly patients (Table S3).

## 4. Discussion

The aim of this study was to assess baseline data, postoperative complications, long-term clinical and radiological outcomes, predictors of neurological improvement and differences between elderly (≥70 years) and non-elderly (18–69 years) patients in a consecutive cohort of 129 patients surgically treated for a spinal meningioma. Previous studies of predictors of outcome in spinal meningiomas have been contradictory and in need of validation from larger cohorts with a long-term follow-up. Schaller et al. reported that tumor size did not influence functional outcomes following spinal meningioma surgery [16], while others found that larger tumors were associated with poor outcomes [2,19]. There are also reports of significant improvement in patients with severe preoperative deficits [18,34], with others suggesting that severe neurological impairment is a limiting factor for complete recovery [17,35]. Three previous studies have also proposed that neurological outcomes can be favorable in the majority of elderly patients, but have been constrained by low patient numbers and the lack of a younger control group [21,22,29]. In this study, we tried to address these conflicting findings in a large cohort with significantly longer follow-up time than previous reports, and with extra emphasis on comparisons between elderly and non-elderly patients.

Compared to the preoperative assessment, surgery was followed by improvement of motor deficits, sensory deficits, gait disturbances, bladder dysfunction and pain. This is largely on par with the few previous studies that have analyzed neurological outcomes after spinal meningioma surgery with a long follow-up and in a large population [36,37]. We also found that time to surgery predicted improvement in mMCs, with an OR of 0.85, indicating that the concept of “time is spinal cord” is applicable to slow growing spinal tumors as well. This suggests that patients with spinal meningiomas should be scheduled for surgery as early as possible. As expected, tumor area (OR 1.73) and spinal cord compression (OR 1.03) also predicted improvement in mMCs in the univariable analysis, indicating that those with a higher degree of spinal cord compression benefited more from surgery. A possible explanation for this might be that increased spinal cord compression leads to more neurological deficits (i.e., pushing the patients higher on the mMCs) but, as long as there is no permanent damage to the cord, patients may improve several steps on the mMCs, postoperatively rendering a larger degree of improvement.

It is of note that we did not find any difference in outcome based on tumor location. This is probably because microsurgery allows all lesions to be treated with minimally traumatic techniques, which allows management without surgical trauma and good outcomes regardless of previously suggested prognostic factors. A higher WHO grade was not associated with a difference in reoperation rate or recurrence. Generally, time to recurrence is expected to be shorter for more aggressive histological tumors following subtotal resection [38]. One explanation for our results is that we only had two cases of WHO grade II tumors, which probably limited the statistical analysis.

Simpson grading is an important predictor of recurrence and progression-free survival in meningiomas [39]. In our cohort, gross total resection (Simpson grade 2 or 3) was achieved in 109 patients (84%). Achieving Simpson grade 1 resection is sometimes difficult, especially in patients with ventral dural attachment, because of the risk of damaging the spinal cord and the difficulty of dural repair. For elderly patients, life expectancy may be shorter than the time needed for a tumor regrowth to cause symptoms. Particularly in these cases, aggressive treatment of the dura to achieve Simpson grade 1 will need to be weighed against the increased risk of neurological complications and cerebrospinal fluid leakage [40]. Our long-term tumor control was more than adequate, with two cases of tumor recurrence following a Simpson grade 2 (*n* = 2) resection (of which one tumor bordered WHO grade 2), and four cases of tumor growth following a Simpson grade 4 resection, which corresponds well with the rates described in the literature [36]. However, recent studies with follow-up times > 10 years suggest that Simpson grade 2 resection may be associated with higher tumor recurrence than previously believed [6,8,9,10,11,41]. Therefore, while tumor control for our cohort was acceptable, it remains unclear whether long-term results would have been better if Simpson grade 1 resections were attempted instead.

In our cohort, which included 20 cases of Simpson grade 4 resection (i.e., subtotal resection), only one patient received adjuvant radiotherapy. Radiation is not usually indicated for WHO grade I meningiomas, although partially resected and high-grade meningiomas have been suggested to benefit from it. However, patients who receive radiation therapy for spinal meningiomas may also develop late onset radiation-induced myelopathy [42]. Moreover, radiation is typically only feasible once and will make any additional surgery more hazardous, and fractionated radiation therapy is not typically curative for meningiomas. In our series, with 20 Simpson grade 4 resections, only four patients experienced tumor progression, two of whom required surgery. Thus, conscientious follow-up and repeated surgery appeared to be a viable alternative to immediate adjuvant radiation, and we had a similarly satisfactory experience in our management of malignant intramedullary tumors without immediate radiation therapy after surgery [43]. However, determining the role of adjuvant radiotherapy for spinal meningiomas was beyond the scope of this study and clinically sound conclusions cannot be made.

Several studies on cranial meningiomas have shown that high age is a risk factor for postoperative pneumonia, postoperative hemorrhage, venous thromboembolism and 30-day mortality [44,45,46,47]. In our cohort, sex distribution, tumor localization, Simpson grade, adjuvant treatment and postoperative complication rates were similar in elderly and non-elderly patients, in line with previously reported data [48]. The rate of tumor recurrence was also similar in elderly and non-elderly patients, which could indicate the similar biological behavior of most (low-grade) meningiomas. This is further supported by the fact that there was no significant difference in histopathological grading (MIB1-index or WHO grade) between the two groups, and it is consistent with previous studies on age-related features of spinal meningiomas [49], but it differs from intracranial meningiomas where MIB1-index values are generally higher in elderly patients [50]. In line with previously published data, the elderly cohort also had a higher degree of preoperative neurological impairment compared to the non-elderly [29]. This finding could be the result of delayed diagnosis due to symptoms being attributed to age-related diseases. If so, investigations of differential diagnoses may have taken time and resulted in the patients being more neurologically affected than the non-elderly group. This is possibly also reflected in the time from diagnosis to surgery, which was significantly shorter in the elderly group and probably due to the presentation of more severe deficits at diagnosis leading to acute or subacute surgery. This observation could also reflect a bias where elderly patients with minor deficits are more likely to be treated conservatively than non-elderly patients with similar symptoms. However, we have no data to support this claim. There were no differences between the elderly and non-elderly in postoperative mMCs improvements or complications, even though the elderly group had a significantly higher ASA class (Table 2 and Table 3, Figure 4). These findings differ from those of an earlier retrospective study of elderly patients with spinal meningiomas, where elderly patients were more likely to experience systemic postoperative complications [29]. Collectively, the above-described findings lend support to the notion that, unlike cranial meningiomas, age may not be a significant predictor of functional outcome or postoperative complications in surgical treatment of spinal meningiomas.

Two patients developed postoperative kyphosis, which could be managed without surgery in both cases. Postoperative kyphosis is a potential complication of open surgery, especially in the cervical spine, and minimally invasive techniques are increasingly implemented in these patients [51,52,53], although there is no evidence that this leads to any significant improvement in outcome [54]. We have previously reported that the incidence of kyphosis following cervical laminectomy and tumor resection is low, with only a few cases requiring later delayed stabilization [55].

### Limitations

This study has several limitations. The retrospective nature does not allow all aspects of the spinal meningiomas to be evaluated. The long follow-up time of 8.2 years may still be too short to evaluate recurrence after Simpson grade II resection, as intracranial meningiomas may recur up to 25 years after surgery [41,56]. The time from diagnosis to surgery is a robust time interval and a predictor of outcome, but the time from first symptoms to diagnosis may be even more important. It is not, however, a readily traceable parameter, especially in elderly patients where symptoms may be misinterpreted for degenerative spine disorders. Moreover, due to underpowered analysis we did not attempt to identify predictors of postoperative complications or tumor growth and recurrence. Partly due to limited availability, neurophysiological monitoring was not used in this study. While we believe neurophysiological monitoring to be an essential part of the set-up for intramedullary spinal tumor surgery, it has a more limited role in extramedullary lesions. The use of intraoperative neuromonitoring could provide a benefit for tumors with anterior location or infiltrative growth, or surgeries where Simpson grade I resection is attempted, since these cases may require more extensive manipulation of the spinal cord.

## 5. Conclusions

In this population-based cohort study of patients treated for spinal meningiomas, surgery was associated with significant neurological improvement. A shorter time from diagnosis to surgery, larger tumor size and spinal cord compression predicted postoperative improvement in mMCs, with spinal cord compression identified as an independent predictor. There was no significant difference in the number of postoperative complications, tumor control or improvement in mMCs between elderly and non-elderly patients.

## Figures and Tables

**Table 1:** Modified McCormick scale explanation.

Grade	Explanation
1	Intact neurologically, normal ambulation, minimal dysesthesia
2	Mild motor or sensory deficit, functional independence
3	Moderate deficit, limitation of function, independent w/external aid
4	Severe motor or sensory deficit, limited function, dependent
5	Paraplegia or quadriplegia, even w/flickering movement

**Table 2:** Baseline data.

Variables	All Patients (*n* = 129)	Elderly (*n* = 46)	Non-Elderly (*n* = 83)	*p*-Value
Age (years)	65 (23–94)	76 (70–94)	58 (23–69)	-
Male sex	23 (18%)	10 (22%)	13 (16%)	0.388
ASA class	2 (1–3)	3 (2–3)	2 (1–3)	**0.023**
Prior radiotherapy	2 (1.6%)	0 (0%)	2 (2.4%)	-
Prior spinal surgery	1 (0.8%)	1 (2.2%)	0 (0%)	-
Concurrent cranial meningioma	8 (6%)	2 (4%)	6 (7%)	-
Neurofibromatosis type 2	1 (0.8%)	0 (0%)	1 (1.2%)	-
Neurological deficits				
Motor deficit	85 (66%)	36 (78%)	49 (59%)	**0.027**
Sensory deficit	82 (64%)	35 (76%)	47 (57%)	**0.028**
Gait disturbance	79 (61%)	35 (76%)	44 (53%)	**0.010**
Bladder dysfunction	45 (35%)	16 (35%)	29 (35%)	0.986
Pain	66 (51%)	19 (41%)	47 (57%)	0.095
Modified McCormick scale (mMCs)	2 (1–4)	3 (1–4)	2 (1–4)	**<0.001**
mMCs 1	33 (26%)	7 (15%)	26 (31%)	-
mMCs 2	47 (36%)	11 (24%)	36 (43%)	-
mMCs 3	37 (29%)	21 (46%)	16 (19%)	-
mMCs 4	12 (9.3%)	7 (15%)	5 (6.0%)	-
mMCs 5	0 (0%)	0 (0%)	0 (0%)	-
Tumor level				
Cervical	39 (30%)	12 (26%)	27 (33%)	0.445
Thoracic	89 (69%)	34 (74%)	55 (66%)	-
Lumbar	1 (0.8%)	0 (0%)	1 (1.2%)	-
Anterior tumor component	30 (23%)	14 (30%)	16 (19%)	0.151
Tumor area (cm^2^)	1.4 (0.2–4.5)	1.4 (0.4–3.1)	1.4 (0.2–4.5)	0.689
Spinal cord compression (%)	69 (12–89)	69 (20–89)	69 (12–89)	0.303

Data presented as median (range) or count (proportion). Bold text indicates a statistically significant correlation (*p* < 0.05). Abbreviations: ASA = American Society of Anesthesiologists; mMCs = modified McCormick scale.

**Table 3:** Treatment data.

Variable	All Patients (*n* = 129)	Elderly (*n* = 46)	Non-Elderly (*n* = 83)	*p*-Value
Time from diagnosis to surgery (months)	1.3 (0.03–36)	0.5 (0.03–36)	1.8 (0.03–30)	**0.005**
Laminectomy range (levels)	3 (1–6)	3 (1–6)	2 (1–5)	0.803
Laminoplasty	35 (27%)	9 (20%)	26 (31%)	0.150
Simpson grade				
Simpson grade 1	0 (0%)	0 (0%)	0 (0%)	-
Simpson grade 2	92 (71%)	32 (70%)	60 (72%)	0.743
Simpson grade 3	17 (13%)	6 (13%)	11 (13%)	0.973
Simpson grade 4	20 (16%)	8 (17%)	12 (14%)	0.194
Simpson grade 5	0 (0%)	0 (0%)	0 (0%)	-
Histopathological data				
Meningioma WHO grade 1	127 (98%)	45 (98%)	82 (99%)	0.670
Meningioma WHO grade 2	2 (1.5%)	1 (2.2%)	1 (1.2%)	-
Meningioma WHO grade 3	0 (0%)	0 (0%)	0 (0%)	-
MIB1-index (%)	4.5 (0–20)	4.8 (1.0–20)	4.0 (0–15)	0.944
Postoperative radiotherapy	1 (0.8%)	1 (2.2%)	0 (0%)	-
Postoperative chemotherapy	0 (0%)	0 (0%)	0 (0%)	-

Data presented as median (range) or count (proportion). Bold text indicates a statistically significant correlation (*p* < 0.05). Abbreviation: WHO = World Health Organization.

**Table 4:** Outcome data.

Variable	All Patients (*n* = 129)	Elderly (*n* = 46)	Non-Elderly (*n* = 83)	*p*-Value
Time to initial follow-up (months)	4.6 (2.7–10)	4.7 (3.2–9.2)	4.4 (2.7–9.8)	0.516
Long-term follow-up time (years)	8.2 (0.3–16)	6.8 (0.3–16)	8.6 (3.1–16)	0.117
Postoperative complication	10 (7.8%)	1 (0.8%)	9 (11%)	0.077
Tethered spinal cord	1	1	0	-
Wound infection	3	0	3	-
Pneumonia	1	0	1	-
Cerebrospinal fluid leak	2	0	2	-
Kyphosis	2	0	2	-
Myocardial infarction	1	0	1	-
Reoperation	4 (3.1%)	2 (4.3%)	2 (2.4%)	0.543
Tethered spinal cord	1	1	0	-
Tumor resection (local tumor recurrence)	2	1	1	-
Wound revision (infection)	1	0	1	-
Tumor growth/recurrence	6 (4.7%)	1 (2.2%)	5 (6.0%)	0.320
Local recurrence	2	1	1	-
Local progression	4	0	4	-
Change in mMCs	0 (−1–3)	0.5 (0–2)	0 (−1–3)	0.629
Improved	61 (47%)	23 (50%)	38 (46%)	0.646
Unchanged	66 (51%)	23 (50%)	43 (52%)	0.844
Worse	2 (1.6%)	0 (0%)	2 (2.4%)	-
Mortality				
3-month mortality	0 (0%)	0 (0%)	0 (0%)	-
Long-term mortality	27 (21%)	22 (48%)	5 (6.0%)	**<0.001**
Tumor-related death	1 (1%)	1 (2%)	0 (0%)	-
Years from surgery to death	6.6 (0.3–15)	5.9 (0.3–15)	8.5 (3.1–12)	-

Data presented as median (range) or count (proportion). Bold text indicates a statistically significant correlation (*p* < 0.05). Abbreviations: mMCs = modified McCormick scale.

**Table 5:** Change in neurological status following surgery.

Postoperative Change	Motor	Sensory	Gait	Bladder	Pain
Patients with preoperative deficit (*n*)	85	82	79	45	66
Completely resolved	39 (46%)	38 (46%)	40 (51%)	34 (76%)	35 (53%)
Unchanged/partial improvement	46 (54%)	44 (54%)	39 (49%)	11 (24%)	31 (47%)
Worse (increased deficit)	0 (0%)	0 (0%)	0 (0%)	0 (0%)	0 (0%)
Worse (new deficit)	2	4	2	2	9
*p*-value (paired testing)	**<0.001**	**<0.001**	**<0.001**	**<0.001**	**<0.001**

Data presented as median (range) or count (proportion). Bold text indicates a statistically significant correlation (*p* < 0.05).

**Table 6:** Univariable logistic regression predicting postoperative improvement in mMCs.

Variable	*p*-Value	OR (95% CI)	Nagelkerke’s R2
Elderly	0.646	-	
Male sex	0.330	-	
ASA class ≥ 3	0.589	-	
Months to surgery	**0.005**	0.86 (0.76–0.94)	0.141
Cervical tumor	0.830	-	
Anterior tumor	0.734	-	
Tumor area (cm^2^)	**0.030**	1.73 (1.00–3.17)	0.053
Spinal cord compression (%)	**<0.001**	1.03 (1.01–1.06)	0.137
MIB1-index	0.053	-	
Simpson grade ≥ 3	0.690	-	

Data presented as median (range) or count (proportion). Bold text indicates a statistically significant correlation (*p* < 0.05). Abbreviations: ASA = American Society of Anesthesiologists; mMCs = modified McCormick scale.

**Table 7:** Final step-down multivariable logistic regression model predicting improvement in mMCs.

Variable	*p*-Value
Included in final step-down model	
Spinal cord compression	**0.002**
Not included in final step-down model	
Months to surgery	>0.05
Tumor area	>0.05

Bold text indicates a statistically significant correlation (*p* < 0.05). Abbreviations: mMCs = modified McCormick scale.

## Data Availability

Data is available from the corresponding author upon reasonable request.

## Supplementary Materials

The following are available online at https://www.mdpi.com/article/10.3390/cancers13133244/s1. Table S1: Previous studies reporting outcomes following spinal meningioma surgery in the elderly, Table S2: Univariable logistic regression predicting postoperative improvement in mMCs in the non-elderly cohort, Table S3: Univariable logistic regression predicting postoperative improvement in mMCs in the elderly cohort.

## Abbreviations

ASA	American Society of Anesthesiologists
mMCs	modified McCormick scale
MRI	magnetic resonance imaging
OR	odds ratio
WHO	World Health Organization
