# Supplementary material for: Long-Term Follow-Up and Predictors of Functional Outcome after Surgery for Spinal Meningiomas: A Population-Based Cohort Study
Source: Cancers (Basel). 2021 Jun 29;13(13):3244. doi: 10.3390/cancers13133244 (PMC8269374; doi:10.3390/cancers13133244)
Supplement: Supplementary file 1 [file cancers-13-03244-s001.zip › cancers-1190674-supplementary.pdf]

Long-Term Follow-Up and Predictors of Functional Outcome after Surgery for Spinal Meningiomas: A Population-Based Cohort Study

Jenny Pettersson-Segerlind, Alexander Fletcher-Sandersjöö, Charles Tatter, Gustav Burström, Oscar Persson, Petter Förander, Tiit Mathiesen, Jiri Bartek, Jr., Erik Edström and Adrian Elmi-Terander

Table S1. Previous studies reporting outcome following spinal meningioma surgery in the elderly.

Reference	Study Design	Definition of Elderly	Elderly Patients	Non-Elderly Control Group	Results
Engel	Retrospective	≥70 years	44	85	All patients improved in mMCs, KPS and mCSS. Surgical complication rates were similar in the elderly non-elderly (6.8 vs. 5.9 %), but systemic complications were more common in the elderly (6.8 vs. 0 %).
Sacko	Retrospective	≥70 years	102 with paraplegia/paraparesis	N/A	91% had improved in their paraplegia or severe paraparesis at 1-year follow-up, of whom 48% recovered completely. 9% experienced a postoperative complication.
Morandi	Retrospective	≥70 years	30	N/A	100% had improved neurologically at 1-year follow-up. 1 patient (3%) experienced a postoperative complication.

Abbreviations: KPS = Karnofsky Performance Status, mCSS = modified Clinical Scoring System, mMCs = modified McCormick scale.

Table S2. Univariable logistic regression predicting postoperative improvement in mMCs in the non-elderly cohort.

Variable	mMCs improved (*n* = 38)	mMCs not improved (*n* = 45)	*p*-value
Time to surgery (months)	0.5 (0.03–10)	5.0 (0.03–30)	**<0.001**
Tumor area (cm²)	1.6 (0.2–4.5)	1.2 (0.3–2.6)	**0.014**
Spinal cord compression (%)	76 (12–89)	56 (17–87)	**0.002**

Data presented as count (proportion). Bold text in the *p* value column indicates a statistically significant correlation ($p < 0.05$). Abbreviations: mMCs = modified McCormick scale.

Table S3. Univariable logistic regression predicting postoperative improvement in mMCs in the elderly cohort.

Variable	mMCs improved (*n* = 23)	mMCs not improved (*n* = 23)	*p*-value
Time to surgery (months)	0.6 (0.03–36)	0.5 (0.03–35)	0.533
Tumor area (cm²)	1.5 (0.7–2.2)	1.4 (0.4–3.1)	0.839
Spinal cord compression (%)	72 (44–89)	66 (20–86)	0.176

Data presented as count (proportion). Abbreviations: mMCs = modified McCormick scale.
